# Triple-Phase Multimodal Knowledge Aggregation Framework for Microbial Keratitis Subtype Diagnosis on Slit-Lamp Photography

**Published:** 2026-07-08

**Authors:** Yiqing Wang, Maria A. Woodward, Ziyun Yang, N. Venkatesh Prajna, Chunming He, Leslie M. Niziol, Mercy Pawar, Ming-Chen Lu, Guillermo Amescua, Rachel Wozniak, Sejal Amin, Abinaya Krishnan, Prabhleen Kochar, Sina Farsiu

**Affiliations:** 1 Department of Biomedical Engineering, Duke University, Durham, NC, USA.; 2 Kellogg Eye Center, Department of Ophthalmology and Visual Sciences, University of Michigan, Ann Arbor, MI, USA.; 3 Department of Cornea and Refractive Surgery Services, Aravind Eye Care System, Madurai, Tamil Nadu, India.; 4 Bascom Palmer Eye Institute, Department of Ophthalmology, University of Miami Miller School of Medicine, Miami, FL, USA.; 5 Flaum Eye Institute, Department of Ophthalmology, University of Rochester Medical Center, Rochester, NY, USA.; 6 Department of Ophthalmology, Henry Ford Hospital, Detroit, MI, USA.; 7 Duke Eye Center, Duke University School of Medicine, Durham, NC, USA.

## Abstract

Microbial keratitis requires rapid pathogen identification to guide treatment, but culture- and PCR-based diagnostics are slow and resource-intensive. We developed a triple-phase multimodal framework for bacterial-versus-fungal keratitis classification using slit-lamp photographs acquired under blue-light, sclerotic-scatter, and white-light illumination, together with clinical metadata. The model combines cross-modality contrastive learning, modality-specific fine-tuning, and feature-level multimodal ensemble learning for patient-level prediction. We evaluated the framework on a multicenter dataset of 1,645 patients and 17,158 images from India and the United States. The model achieved 85.84% accuracy, 84.46% average F1-score, and 0.885 AUC. Site-specific evaluation showed that pooled results were overly optimistic, whereas resampling- and balance-based re-evaluation provided a more realistic assessment of cross-site generalization. Under all settings, our framework remained the top-performing approach. The code is available at https://github.com/yqwang01/TPMKA and dataset access will be provided subject to University of Michigan data-sharing clearance.

## 2. Introduction

Microbial keratitis (MK) is a serious corneal infection that can lead to vision impairment, severe pain, and reduced productivity^1^. A major challenge in managing MK lies in the absence of a reliable, rapid, and efficient method to identify the causative pathogen at the time of diagnosis — a crucial factor to obtaining targeted treatment and preventing vision loss^2–4^. Current diagnostic methods^5,6^, primarily based on culture from corneal scrapings, fail to identify the pathogen in over 50% of cases. These cultures require several days to yield results, and point-of-care testing options are unavailable. The inability to pinpoint the causative organism often leads to non-specific and suboptimal treatment, resulting in poorer clinical outcomes, avoidable side effects, and increased costs for both patients and healthcare systems^7–9^. Therefore, there is an urgent need to develop accessible, practical tools that enable rapid identification of MK pathogens and facilitate timely communication of results to eye care professionals^10^.

Slit-lamp photography (SLP)^11^ is a high-resolution ocular imaging technique that is widely adopted due to its low cost, high image quality, and ease of operation. Using a light source and a light filament, ophthalmologists can adjust the length and width of the circular light beam and magnify the image up to 40×. SLP enables the visualization of specific clinical biomarkers of MK—such as stromal infiltrates and hypopyon—under varying illumination conditions^12,13^. Blue light, often combined with fluorescein staining, highlights corneal surface damage, while sclerotic scatter reveals intracorneal changes like opacification and edema, and white light offers a comprehensive view of ocular anatomy. These light sources enhance clinicians’ diagnostic accuracy by illuminating specific features, increasing lesion contrast, and enabling multi-angle assessment. An automatic algorithm for processing slit-lamp images has the potential to assist clinical decision-making and improve treatment strategies for MK.

In recent years, deep learning technologies have gained widespread adoption across various fields, demonstrating remarkable performance in disease detection. As such, previous studies have leveraged deep neural networks to classify MK into subtypes, such as bacterial and fungal infections, based on slit-lamp images^14–27^. However, these approaches often face limitations in clinical applicability, as they are typically developed using data from a single medical center, and often exhibit suboptimal performance on external cohorts. Moreover, naïvely combining multi-center data for training^18,23^ may lead to overly optimistic performance estimates, as it can introduce overfitting and amplify biases arising from differences in data distributions across centers.

Diagnosing MK subtypes using slit-lamp images poses several key challenges: 1) The subtle visual differences between bacterial and fungal infections are often imperceptible. Even experienced ophthalmologists may struggle to accurately distinguish pathogens based solely on slit-lamp images. 2) Significant heterogeneity in slit-lamp images—resulting from varying illumination modalities and magnification levels—creates substantial obstacles for feature extraction. Deep neural networks are prone to focusing on superficial characteristics while neglecting essential lesion-related features. 3) The scarcity of large-scale, annotated SLP datasets limits the generalization and reliability of deep learning models.

To address these challenges, we propose a novel machine learning algorithm for MK pathogen classification. We curated a large-scale, multi-center, multi-modality dataset comprising slit-lamp images from over 1,600 patients with bacterial or fungal MK. To fully leverage the rich information provided by different imaging conditions, we design a triple-phase framework that processes images captured under diverse illumination and magnification settings. We introduce a contrastive learning-based^28^ method to extract robust, modality-invariant features across varying data patterns. These features are subsequently integrated across modalities to facilitate the final prediction. Building upon our previous work^29,30^, which developed an effective segmentation network for identifying key biomarkers of MK in slit-lamp images, we apply limbus-based cropping to remove irrelevant background regions and reduce noise. Additionally, we incorporate metadata representing clinical context, which further enhances model performance. Extensive experiments demonstrate that our approach consistently outperforms representative baselines and achieves state-of-the-art performance in MK subtype classification, reaching 85.84% accuracy, 84.46% average F1-score, and 0.885 AUC. Moreover, site-specific analyses reveal that naïve evaluation on pooled multi-center data can be overly optimistic, whereas our framework remains robust under more stringent resampling and balanced evaluation settings, highlighting its stronger potential for fair and generalizable cross-site deployment.

### Our main contributions are threefold:

- We construct **a large-scale, multi-center, SLP dataset** for MK, comprising 17,158 images from 1,645 patients (1,048 fungal and 597 bacterial cases) collected in the United States and India. The dataset contains up to three illumination modalities per patient— diffuse blue light with fluorescein staining, sclerotic scatter, and diffuse white light—together with paired clinical metadata, including trauma history, organic-matter and water exposure, contact-lens use, and visual acuity. Although the dataset cannot be publicly released due to institutional patient privacy constraints, access may be provided upon reasonable email request and subject to appropriate approvals and data-use agreements with University of Michigan.
- We propose a **triple-phase multi-modality framework** for MK subtype classification that explicitly addresses the heterogeneity of real-world slit-lamp imaging: Phase One uses Cross-Modality Contrastive Learning (CMCL) to learn modality-invariant representations by treating images of the same patient — regardless of illumination or magnification — as positive pairs; Phase Two fine-tunes three modality-specific networks on top of the CMCL-pretrained weights to capture illumination-specific cues; and Phase Three integrates the three modality embeddings through a multi-layer perceptron (MLP)-based feature ensemble that gracefully handles missing modalities via zero-vector initialization. The framework is further strengthened by limbus-based cropping of the images (building on our prior segmentation work) and tabular metadata fusion, and achieves state-of-the-art performance (85.84% accuracy, 84.46% average F1, 0.885 AUC), with the largest gains on the historically difficult bacterial class (F1 improved from 76.7% to 80.0%).
- Through **site-stratified evaluation under three complementary protocols** — training on the original distribution, site- and class-weighted resampling, and a separately curated fully balanced subset — we expose and quantify a pitfall that has been largely overlooked in prior multi-center keratitis studies: aggregate metrics on pooled data are **overly optimistic** and mask pronounced per-site, per-class weaknesses (e.g., bacterial F1 as low as 39.8% on the India subset and fungal F1 as low as 31.0% on the US subset for strong baselines). A controlled “No Location” ablation further shows that this geographic bias persists even when the site variable is removed from the metadata, suggesting that the network can recover site identity from image-level cues alone. Our framework consistently ranks first across all three protocols, providing what we believe is a more faithful benchmark for cross-site generalization in AI-assisted keratitis subtype diagnosis.

## 3. Results

### 3.1. Model Performance Evaluation and Comparison

**Figure 1.**
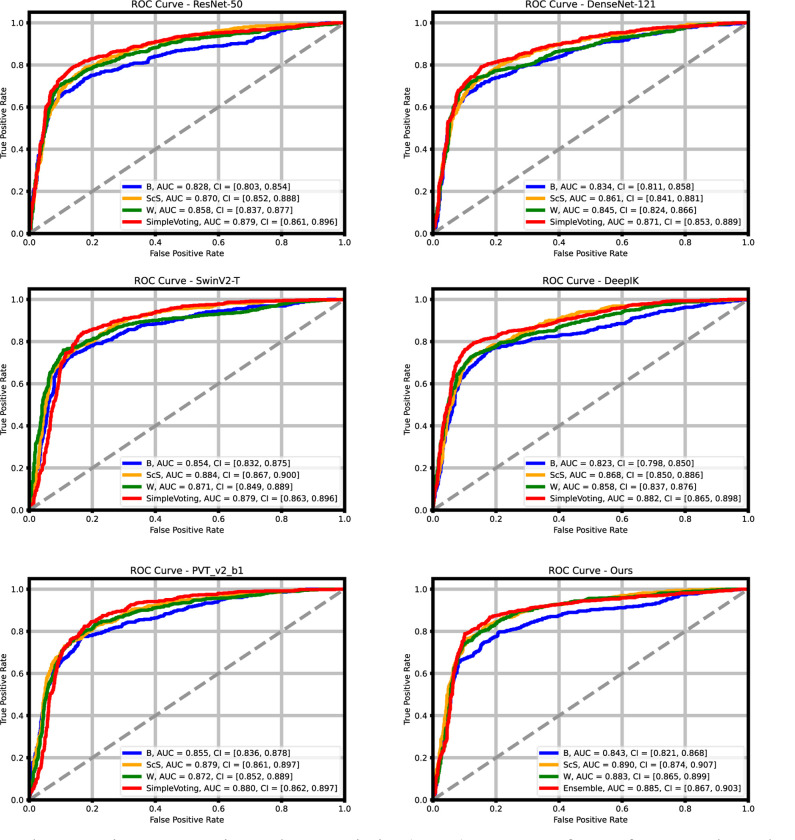
The Receiver Operating Characteristic (ROC) curves of our framework and other baselines. B – Blue Light, ScS – Sclerotic Scatter, W – White Light.

We conducted extensive experiments on our framework, comparing its performance against several established baselines, including DeepIK^23^, ResNet-50^31^, DenseNet-121^32^, SwinV2-T^33^, and PVT_v2_b1^34^. The outcomes of these experiments are summarized in Table 1. In our framework, we adopt PVT_v2_b1^34^ as the backbone. We report five-fold cross-validation results, including mean F1-scores, accuracy, standard deviations, and ROC-AUCs in Table 1 and Figure 1.

For consistency in evaluation, we follow the general approach that trains the baseline methods on each modality separately. All methods followed the same preprocessing pipeline, including image augmentation, limbus region cropping, and incorporation of metadata. When a patient had more than one image per modality, we obtained the predictions for that modality using a simple majority voting mechanism. For every baseline, we obtained the final predictions with a simple majority voting across three modalities. In contrast, our framework integrates an additional ensemble module that aggregates features from multiple images of different modalities, resulting in notable performance improvements, as indicated in Table 1.

The results indicate that the main challenge lies in bacterial classification. While most baseline models achieved relatively high fungal F1-scores, generally around 87%–89%, their bacterial F1-scores were consistently lower, mostly ranging from 66.8% to 76.7%. This gap suggests that the imbalanced data distribution has a stronger adverse effect on bacterial recognition. Compared with these baselines, our framework substantially improved bacterial classification performance. In particular, the ensemble version increased the bacterial F1-score to 80.0%, while maintaining a high fungal F1-score of 89.0%, resulting in the best average F1-score of 84.46%.

Among the single-modality settings, our framework already showed competitive or superior performance. Using the diffuse blue light illumination, our framework achieved an average F1-score of 78.76% with 83.28% accuracy, comparable to the strongest baselines. Under the sclerotic scatter illumination, our framework achieved the best performance among all methods, reaching 75.4% bacterial F1, 82.12% average F1, and 84.81% accuracy. Under diffuse white light illumination, our framework also performed strongly, yielding 76.7% bacterial F1 and 82.36% average F1. These results suggest that our framework consistently improves the more difficult bacterial category across different illumination modalities, with especially strong gains under sclerotic scatter illumination and diffuse blue light illumination.

Overall, our framework achieved the best performance among all compared methods. With PVT_v2_b1^34^ as the backbone, the proposed ensemble model reached an accuracy of 85.84% and an average F1-score of 84.46%, outperforming all baselines, including the strongest baseline PVT_v2_b1^34^ with simple voting (85.05% accuracy and 82.80% average F1) and SwinV2-T^33^ with simple voting (84.74% accuracy and 82.39% average F1). Pairwise comparisons between the proposed framework and each baseline were performed using two-sided Wilcoxon signed-rank tests on the five fold-level cross-validation results (n = 5), with Holm correction applied for multiple comparisons and statistical significance assessed at α = 0.05; the proposed framework showed statistically significant improvements over the compared baselines after correction.

The ROC analysis further supports the superiority of our framework. Among the compared methods, our approach achieved the highest AUC values in both the sclerotic scatter and white-light modalities, reaching 0.890 (95% CI: 0.874–0.907) and 0.883 (95% CI: 0.865–0.899), respectively. Although the blue-light modality showed a relatively lower AUC of 0.843 (95% CI: 0.821–0.868), the proposed ensemble module further improved the overall discriminative ability, producing an AUC of 0.885 (95% CI: 0.867–0.903).

Together, these results demonstrate that our framework not only achieves the best overall performance but also addresses the key weakness of existing methods, namely, the limited recognition ability for bacterial cases. The consistent gains across F1-score, accuracy, and AUC indicate that the proposed multimodal ensemble strategy provides a more robust and effective solution for MK subtype classification.

### 3.2. Visualization and Interpretation of Model Outcomes

**Figure 2.**
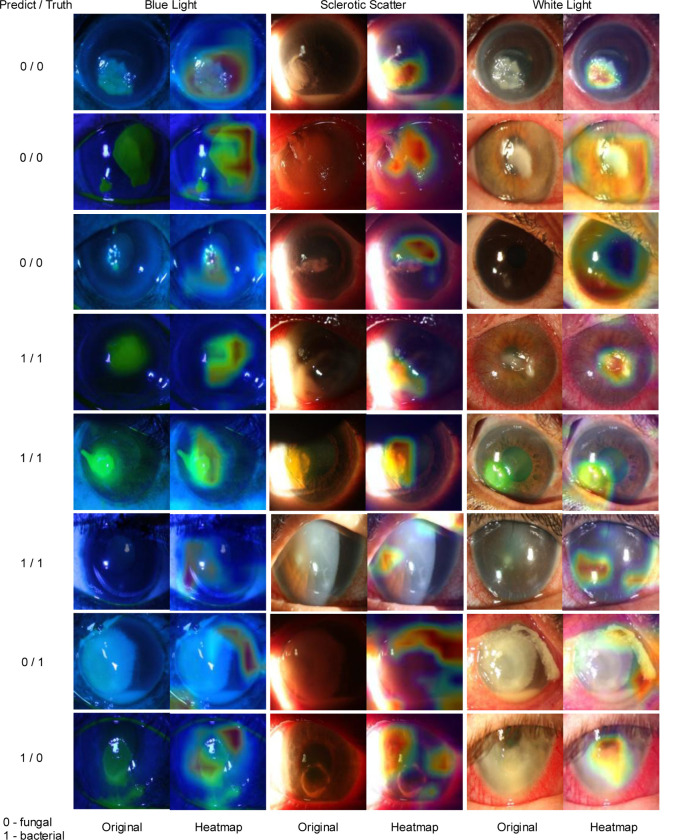
Gradient-weighted Class Activation Maps (Grad-CAM maps) on our framework with the backbone of PVT_v2_b1.

Figure 2 presents representative examples of Grad-CAM maps generated by our framework. Red heatmap areas indicate areas where the model assigns higher attention during decision-making. Our analysis shows that the model frequently focuses on clinically relevant lesion areas, including stromal infiltrates, hypopyon, epithelial defects, and corneal inflammation. However, in cases of incorrect predictions, the highlighted regions sometimes correspond to less relevant anatomical areas such as the sclera or eyelid margin.

### 3.3. Model Component Assessments by Ablation Studies

We conducted comprehensive experiments to investigate the effects of the components in our framework. All these outcomes demonstrate that each element contributes to the final model performance.

#### 3.3.1. The Effects of Cross-Modality Contrastive Learning

We evaluated the impact of CMCL on SwinV2-T^33^ and PVT_v2_b1^34^, and observed consistent performance improvements across all of them, as shown in Table 2. The pretrained parameters from CMCL effectively capture modality-consistent features, which complement and enhance the modality-specific fine-tuning process. Additionally, the ensemble module leverages these features to uncover richer information, enabling the model to achieve higher overall performance when utilizing CMCL.

#### 3.3.2. The Effects of Cropping Limbus Areas

We evaluated the impact of cropping limbus areas on model performance using PVT_v2_b1 as the backbone. As shown in Table 3, this approach significantly enhances performance by directing the model's attention to lesion regions while effectively minimizing the influence of background noise.

#### 3.3.3. The Effects of Metadata Usage

We assessed the effect of incorporating clinical metadata on model performance using PVT_v2_b1 as the backbone. As shown in Table 3, the inclusion of these metadata variables provides complementary information, leading to further improvements in the model's overall performance.

#### 3.3.4. The Effects of Regularization in Fine-tuning

During fine-tuning, the model may face overfitting issues. We observed that incorporating the contrastive learning loss function as a regularization term effectively mitigates this problem. As shown in Table 3, the model achieved improved performance when the regularization weight λ in Eq 3 was set to 0.1.

### 3.4. Site-specific Performance Evaluation

Although the overall results in Table 1 appear encouraging when evaluation is performed without distinguishing geographic sites, such aggregate metrics can be overly optimistic because they obscure substantial distribution shifts across sites. To better understand whether the learned models generalize fairly across domains, we further report site-specific performance in Table 5 under three training settings: original, resampling, and balanced. Here, original denotes direct training on the raw dataset, and resampling refers to weighted sampling across both sites and classes such that the sampled training distribution becomes fully balanced. In addition, we constructed a separate balanced dataset containing equal numbers of patients from each site and class for controlled evaluation. The corresponding class distributions are summarized in Table 4. By comparing these three settings, we aim to assess not only the overall classification performance but also the extent to which data imbalance across sites and classes affects model fairness and robustness.

Under the original setting, substantial site-specific disparity was observed. When testing on the India subset, all methods achieved high fungal F1 scores (above 91%), but bacterial recognition remained much weaker, with bacterial F1 scores ranging from 39.8% to 51.9%. In contrast, when testing on the US subset, bacterial classification was consistently strong (87.3%–91.6%), whereas fungal recognition dropped sharply to 31.0%–45.9%. These results show that the favorable overall performance on the original dataset masks clear weaknesses on specific site–class combinations. Notably, this imbalance persisted even after removing explicit clinical-center information. The Ours (No Location) variant still showed the same site-dependent trend: on the India subset, fungal classification remained strong (91.6%) while bacterial F1 was much lower (46.0%), whereas on the US subset, bacterial F1 stayed high (90.9%) but fungal F1 dropped to 37.7%. Its average F1-score was 68.79% on India and 64.31% on the US subset, indicating that the geographic bias was not eliminated by excluding the clinical-center variable. This finding suggests that site-related distribution shifts can still be inferred from the images themselves and/or from other metadata variables, thereby continuing to drive location-dependent prediction bias. Despite this strong domain asymmetry, our framework still achieved the best overall trade-off. Specifically, on the India subset, our framework improved the average F1 score to 71.79%, outperforming DeepIK^23^ (66.95%) and PVT_v2_b1^34^ (65.97%), mainly through better bacterial recognition. On the US subset, our framework achieved the highest bacterial F1 (91.6%) and the best accuracy (85.44%), with an average F1 of 67.27%, comparable to PVT_v2_b1^34^ (67.77%) and substantially higher than DeepIK^23^ (59.16%). When evaluated on the overall original dataset, our framework also obtained the best performance, reaching 84.46% average F1 and 85.84% accuracy. Notably, the overall accuracy was computed by averaging the fold-wise accuracies across five cross-validation folds. Because the site and class compositions varied slightly across folds under random splitting, the averaged overall accuracy is not strictly constrained to lie between the averaged India and US accuracies.

Under the resampling setting, where training samples were reweighted to equalize both site and class frequencies without explicitly discarding data, site-specific performance became more balanced, and minority-domain as well as minority-class recognition improved. In the India subset, our framework achieved the best average F1 of 73.74%, slightly exceeding PVT_v2_b1^34^ (73.29%) and DeepIK^23^ (72.18%), while also obtaining the highest bacterial F1 (57.4%). In the US subset, the improvement was more evident: our framework increased fungal F1 to 49.6% and achieved the best bacterial F1 (90.5%), resulting in the highest average F1 (70.06%) and accuracy (84.12%), outperforming both baselines by a clear margin. At the overall level, our framework again ranked first, with 83.03% average F1 and 84.19% accuracy. These findings suggest that resampling can partially alleviate training bias while preserving more of the original data diversity than strict subsampling.

Under the fully balanced setting, where the dataset was constructed to contain equal numbers across sites and classes, performance across India and the US became markedly more consistent than in the original setting. Our framework achieved the best results across all evaluation splits. In the overall evaluation, our framework reached 75.44% average F1 and 75.59% accuracy, outperforming DeepIK^23^ (73.96% / 74.43%) and PVT_v2_b1^34^ (72.81% / 73.29%). On the India subset, our framework again achieved the strongest performance, with 79.70% average F1 and 80.24% accuracy, exceeding DeepIK^23^ (77.64% / 78.63%) and PVT_v2_b1^34^ (74.77% / 75.57%). On the US subset, our framework also remained superior, reaching 71.36% average F1 and 71.52% accuracy, compared with 69.40% / 70.24% for DeepIK^23^ and 70.00% / 70.70% for PVT_v2_b1^34^. Overall, the reduced gap between India and US performance indicates that balancing the data distribution effectively mitigates geographic bias and provides a more realistic assessment of cross-site generalization.

Overall, these results confirm that evaluation directly on the original dataset is indeed overly optimistic, as high aggregated performance can conceal pronounced weaknesses in certain geographic domains and class combinations. Re-evaluating the models with resampling and balanced training reveals a more realistic picture of site-specific generalization. Although our framework consistently achieves the strongest and most stable performance across all three settings, the remaining discrepancies also indicate that fairness across domains is not fully resolved. In future work, we will explore additional strategies beyond data balancing to promote more equitable training and prediction behavior across different geographic populations.

## 4. Discussion

Previous studies on MK organism subtype classification have typically employed conventional convolutional neural networks to build classification models. However, these approaches often yield suboptimal performance due to their limited capacity to capture latent representations and a lack of task-specific architectural design tailored to slit-lamp imaging scenarios. DeepIK^23^ introduced a novel deep learning framework incorporating a secondary classifier to emulate the diagnostic reasoning of human experts. Nevertheless, it still fails to effectively distinguish between bacterial and fungal keratitis.

To the best of our knowledge, this is among the first studies to introduce contrastive representation learning for MK organism diagnosis from SLP. Our proposed triple-phase framework integrates information from multiple modalities and demonstrates superior performance, flexibility, and robustness in our experiments. In Phase One, a universal model is trained to extract modality-invariant feature representations using CMCL. The resulting pretrained parameters are then transferred to three separate networks in Phase Two for modality-specific fine-tuning, allowing each model to focus on the unique characteristics of its corresponding modality. In Phase Three, features from all modalities are fused using a MLP, which enables flexible handling of varying numbers of image inputs and ensures stable performance across conditions.

We constructed a large-scale, multi-center slit-lamp photography dataset comprising more than 1,600 patients and over 17,000 images. Extensive five-fold cross-validation experiments demonstrated that our framework consistently outperformed representative baselines, including DeepIK^23^, ResNet-50^31^, DenseNet-121^32^, SwinV2-T^33^, and PVT_v2_b1^34^. Using PVT_v2_b1 as the backbone, our final ensemble model achieved the best overall performance, with 85.84% accuracy, 84.46% average F1-score, and 0.885 AUC. Importantly, the main advantage of our framework lies in improved bacterial recognition. While most baseline methods achieved relatively high fungal F1-scores (approximately 87%–89%), their bacterial F1-scores remained substantially lower, generally ranging from 66.8% to 76.7%. In contrast, our ensemble increased the bacterial F1-score to 80.0%, while maintaining a fungal F1-score of 89.0%. Among the single-modality settings, our framework also achieved the strongest performance under sclerotic scatter illumination and diffuse white light illumination, and the ensemble further improved performance beyond conventional majority voting, indicating the benefit of feature-level multimodal fusion.

At the same time, our site-specific experiments revealed that evaluation on the original dataset without separating geographic domains can be overly optimistic. Although the aggregate results appear strong, stratified analysis showed substantial performance disparity across sites. Under the original setting, all methods achieved high fungal F1-scores on the India subset (above 91%), but bacterial recognition remained weak, with bacterial F1 as low as 39.8%–51.9%. In contrast, on the US subset, bacterial classification was consistently strong (87.3%–91.6%), whereas fungal recognition dropped sharply (31.0%–45.9%). Despite these challenges, our framework still achieved the best overall trade-off, reaching 71.79% average F1 on India and 67.27% on the US subset. To further examine this issue, we introduced two additional evaluation settings: resampling, which reweights samples to equalize site and class frequencies, and balanced, which constructs a fully balanced dataset through random subsampling. Under both settings, site-specific performance became more consistent, and our framework remained the top-performing method. In the resampling setting, our framework achieved 83.03% overall average F1, with particularly clear gains on the US subset (70.06% average F1). In the balanced setting, our framework again performed best, reaching 75.44% overall average F1 and 75.59% accuracy, while reducing the gap between India and US performance. These findings indicate that the strong results obtained on the original dataset indeed mask fairness-related issues, and that a more balanced evaluation provides a more realistic view of model generalization across geographic domains. Although clinical-center metadata may improve performance in population-matched deployment settings by reflecting local epidemiological priors, it can also increase the risk of shortcut learning when the model is transferred to new geographic regions. To explicitly assess this risk, we evaluated a No Location variant and conducted site-stratified analyses.

Note that after removing explicit clinical-center metadata (i.e., the No Location ablation), our algorithm still exhibited what appears to be overly optimistic aggregate performance. This suggests that the network may have automatically learned non-disease-related visual cues, such as site-specific imaging signatures (e.g., sensor-dependent noise patterns, illumination color temperature, racial differences in populations between India and the US, or image compression artifacts), to implicitly identify the source institution. Consequently, the model can exploit the underlying epidemiological imbalances present in the training data as a classification shortcut during inference. In clinical practice, reliance on such implicit geographic priors can be either appropriate or inappropriate depending on the deployment context. For instance, if the diagnostic tool is deployed exclusively within the specific clinical populations on which it was trained, leveraging local disease prevalence may legitimately improve diagnostic accuracy. Conversely, if the algorithm is introduced to a novel demographic region (e.g., a European cohort) with fundamentally different baseline pathogen distributions, its expected performance would likely degrade due to domain shift. While curating a globally representative dataset spanning all populations is beyond the scope of this study, our analysis highlights a critical vulnerability regarding geographic bias and shortcut learning—a pitfall that is frequently overlooked when multi-center datasets are pooled and evaluated without site-stratified analysis.

To enhance model interpretability, we visualized Gradient-weighted Class Activation Maps (Grad-CAM maps)^35^, which illustrate the decision-making focus of the models. These visualizations show that the model often attends to clinically relevant lesion areas, though in some misclassified cases, attention is diverted to irrelevant background regions—indicating potential avenues for further improvement. We also performed a series of ablation studies, which validated the necessity and effectiveness of each component within our framework.

Despite the strong performance and multi-center validation of our proposed framework, several limitations warrant consideration. First, although our framework achieves state-of-the-art offline performance on a large retrospective dataset, prospective validation during real-world, point-of-care clinical workflows remain essential before such an AI-driven diagnostic decision-aid tool can be safely deployed to assist eye care clinicians. Retrospective performance on curated images, even across multiple centers, does not fully capture the variability of bedside image acquisition, photographer skill, triage workflow, or the downstream effect of model predictions on clinician decision-making. A prospective, reader-in-the-loop study, ideally in clinical situations with diagnostic uncertainty where rapid pathogen identification would have the greatest clinical impact, is the logical next step.

Second, the current model formulates MK organism diagnosis strictly as a binary classification task (bacterial versus fungal). In clinical reality, keratitis can also be caused by viral pathogens (e.g., Herpes simplex), parasitic organisms (e.g., Acanthamoeba), or present as complex polymicrobial infections. Expanding the label space to cover these additional etiologies, and ideally to support a “non-infectious” or “indeterminate” output rather than forcing a binary call, is a necessary next step for clinical readiness.

Third, our dataset exhibits a pronounced geographical and epidemiological skew, with the Indian center predominantly contributing fungal cases and the US centers primarily providing bacterial cases. Our site-stratified evaluation (Section 3.4) was specifically designed to expose and partially mitigate this bias through resampling and balanced protocols, and our framework remained the top performer across all three settings. Nevertheless, the “No Location” ablation showed that site-dependent cues can still be recovered from the images themselves, indicating that data balancing alone is insufficient. Future work should explore techniques such as domain-adversarial training, site-invariant representation learning, and federated learning across a broader geographic footprint (e.g., European, African, and East Asian cohorts) to rigorously decouple geographic signatures from disease-relevant features.

Fourth, owing to pragmatic constraints in collecting large-scale clinical data, imaging protocols were not strictly adhered to in all cases, resulting in missing illumination modalities for some patients. While our Phase-Three ensemble accommodates missing data via zero-vector initialization, the absence of complete triple-modality image sets inherently limits the diagnostic synergy of the framework. Standardized imaging protocols in future prospective cohorts would enable tighter evaluation of each modality's incremental contribution.

Finally, as highlighted by our Grad-CAM analysis, the network occasionally anchors clinically irrelevant background regions or imaging artifacts in misclassified cases, indicating room for improved spatial attention and noise suppression. Incorporating explicit lesion-level supervision, attention regularization, or uncertainty-aware prediction mechanisms may further improve both accuracy and interpretability.

In summary, we developed a contrastive-learning-based, triple-phase, deep learning framework for classifying bacterial and fungal keratitis from multi-modality slit-lamp photography images. Evaluated on a large multi-center dataset collected from geographically diverse hospitals, our framework consistently outperformed representative baseline methods. Using PVT_v2_b1 as the backbone, the final ensemble model achieved the best overall performance, with 85.84% accuracy, 84.46% average F1-score, and 0.885 AUC. In particular, our framework substantially improved the more challenging bacterial classification, increasing the bacterial F1-score to 80.0% while maintaining strong fungal performance. Further site-specific analyses showed that evaluation on the original dataset alone could be overly optimistic, as substantial geographic disparities were observed after stratifying by site. By re-evaluating the model under resampling and balanced settings, we found that our framework remained the top-performing method and yielded more consistent results across sites, highlighting both its robustness and its potential for fairer cross-domain generalization. In future work, we will further improve the framework to better address misclassified cases and explore additional strategies beyond data balancing to enhance fairness and generalizability across broader and more diverse populations.

## 5. Methods

### 5.1. Dataset Development

**Figure 3.**
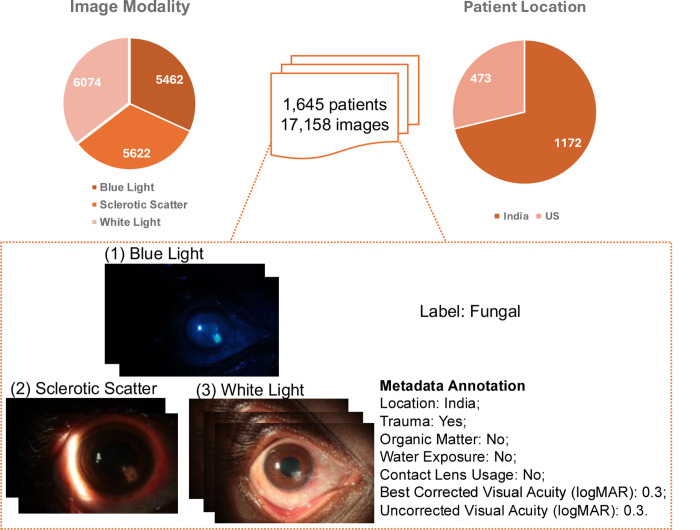
The dataset distribution and a case example featuring three SLP imaging modalities: (1) diffuse blue light illumination (B) following topical fluorescein staining, (2) sclerotic scatter illumination (ScS), (3) diffuse white light illumination (W), and corresponding metadata.

Our study incorporates data that retrospectively identified MK cases using their electronic health records. The participating centers include four hospitals from the US and one hospital from India. The dataset comprises 17,158 slit-lamp images from 1,645 subjects. These subjects consisted of 1,048 patients diagnosed with fungal keratitis and 597 patients diagnosed with bacterial keratitis. Patients were diagnosed by expert classification using the gold-standard culture and PCR-based methods; for culture-negative cases, diagnoses were determined through expert chart review and consultation with the treating clinician. This methodology was used to ensure culture-negative cases were included in the modeling. If cases could not be classified during expert review, they were not included in the dataset. Up to three SLP imaging illumination modalities were acquired for each patient: (1) diffuse blue light illumination (B) with topical fluorescein staining, (2) sclerotic scatter illumination (ScS), and (3) diffuse white light illumination (W). Figure 3 shows a representative image set from one subject. Note that as our study reflects data collected from real-world clinical practice, some imaging sessions capture multiple images for certain SLP illumination settings, while others may not include images for a specific modality of a given subject. The details of the data collected are shown in Table 6. For each patient, we also collected metadata information, including location, contact lens usage, and a series of clinical conditions shown in Table 7. The study protocol was reviewed and approved by the University of Michigan Institutional Review Board (IRB #- HUM00174923 Automated Quantitative Ulcer Analysis study - AQUA study) and the Duke University Institutional Review Board (Protocol ID: Pro00064195), and the study was conducted in accordance with the tenets of the Declaration of Helsinki. All slit-lamp images and associated clinical metadata were de-identified prior to analysis to ensure patient confidentiality. The requirement for informed consent was waived by the ethics committees because the study involved analysis of de-identified clinical data and posed minimal risk to participants.

### 5.2. Framework Overview

**Figure 4.**
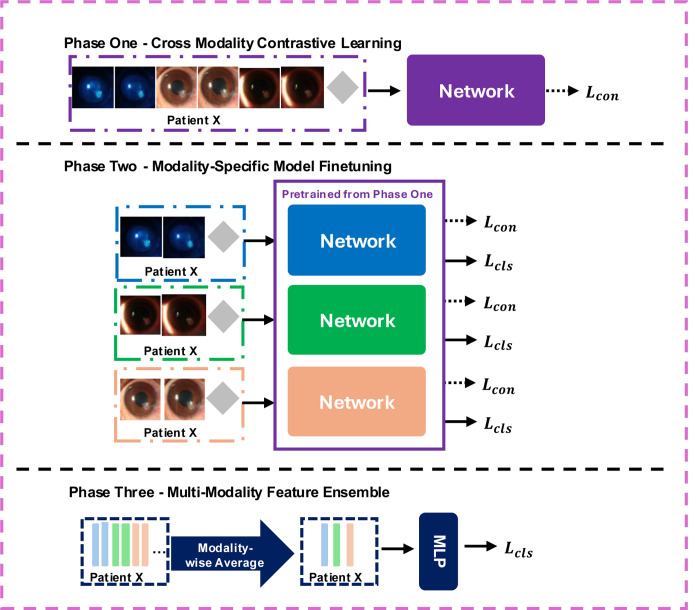
Overview of our triple-phase framework and training procedure for MK subtype classification. For each patient, multiple slit-lamp images were captured under up to three different illumination conditions (modalities), each exhibiting varying acquisition settings. Patient X represents a specific random patient in our dataset. In Phase One, we pretrained our network using Cross-Modality Contrastive Learning (CMCL) to learn general latent features across different images of the same patients. In Phase Two, the pretrained parameters were fine-tuned for each of the three modalities, allowing the model to extract modality-specific representations. Finally, in Phase Three, a Multi-Layer Perceptron (MLP) was used to integrate the latent vectors from the three modality-specific networks and produce the final predictions. The feature vectors corresponding to different modalities are illustrated as rectangles in distinct colors, representing modality-specific representations.

**Figure 5.**
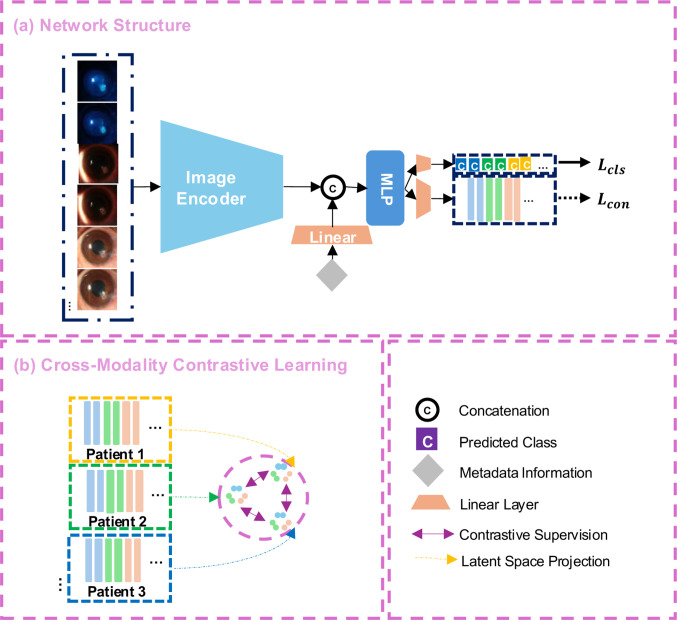
The details of our network. (a) Network structure. The image encoder extracts robust features from slit-lamp images. A linear layer is used to embed the clinical metadata into a feature vector. The image and metadata features are concatenated and fed into a Multi-Layer Perceptron (MLP) for joint representation learning. Subsequently, a projection layer maps the resulting feature vector into a latent space for contrastive learning, while an additional linear layer is employed for the final classification task. (b) Cross-Modality Contrastive Learning. A projection layer maps the fused feature vector (depicted as a filled rectangle) into a latent space, where its position is represented as a circle. Contrastive loss is applied to encourage latent features from the same patient to cluster closely, while pushing those from different patients farther apart within the latent space.

We present a triple-phase framework for MK subtype classification to integrate features from different imaging modalities and metadata. For each patient, multiple images were captured under varying illumination modalities, such as diverse magnification factors including 6.3×, 10×, and 16×. In Phase One, we pretrained our network using CMCL (Section 5.5), to learn general latent features across different images, reducing the risk of overfitting. Subsequently, in Phase Two, the pretrained parameters were fine-tuned for each of the three modalities, allowing the model to extract modality-specific representations (Section 5.6). Finally, in Phase Three, a MLP was used to integrate the latent vectors from the three modality-specific networks and produce the final predictions (Section 5.7). We utilized a connectivity-based segmentation network previously developed by our team^29,30,36,37^ to isolate and crop the limbus regions, effectively reducing noise in the data (Section 5.3). We selected the widely used Pyramid Vision Transformer (PVT)^34,38^ as the backbone architecture. Additionally, metadata were incorporated into the backbone to provide complementary information and enhance the classification performance (Section 5.4). The entire process of this triple-phase framework is illustrated in Figures 4 and 5.

### 5.3. Image Pre-processing

To suppress background noise and guide the model’s attention toward MK lesion-relevant regions, we generated bounding boxes around the limbus. The limbus is the anatomical boundary between the cornea and the sclera, serving as a useful landmark for localizing the corneal region in slit-lamp images. To achieve this, following our previous work^29^, we used a connectivity-based segmentation method, the Bilateral Connectivity Network (BiconNet)^30^, which leverages connectivity masks alongside saliency masks as supervisory signals to effectively model interpixel relationships and object saliency. These bounding boxes are then used to crop the central region of each slit-lamp image before further processing and model training. We demonstrate the effectiveness of limbus region cropping in Section 3.3.2. Additionally, a range of image augmentation techniques, including random cropping, flipping, rotation, and color jittering, are applied to improve the model's robustness.

### 5.4. Backbone Selection and Metadata Usage

We evaluated the performance of four widely adopted deep networks to compare their effectiveness in our task and validate the generalizability of our framework. ResNet-50^31^ and DenseNet-121^32^ represent two classical convolutional neural network (CNN)-based architectures, while the Swin-Transformer^33,39^ and Pyramid Vision Transformer (PVT)^34,38^ employ Transformer-based progressive shrinking structures to efficiently reduce feature size and extract multi-scale representations. Comprehensive experiments conducted with all these backbones demonstrated the efficiency of our framework, with PVT_v2_b1 achieving the best results (shown in Table 1).

In addition to imaging data, clinical metadata were recorded during image collection. Building on insights from our previous study^4^, we integrated seven metadata variables into the network: best-corrected visual acuity of the affected eye, uncorrected visual acuity of the affected eye, trauma history, exposure to organic materials, location, water exposure, and contact lens usage. The contributions of these metadata to the model's performance are detailed in Section 3.3.3.

Finally, we adopted PVT_v2_b1 as the image encoder to extract robust features from slit-lamp photographs. A linear layer is used to embed the clinical metadata into a feature vector. The image and metadata features are concatenated and fed into a Multi-Layer Perceptron (MLP) for joint representation learning. Subsequently, a projection layer maps the resulting feature vector into a latent space for contrastive learning, while an additional linear layer is employed for the final classification task.

### 5.5. Phase One: Cross-Modality Contrastive Learning

Contrastive learning^28^ is a widely adopted technique for enhancing deep networks, bridging gaps across diverse data domains and modalities. It minimizes the distance between samples from the same case and maximizes the disparity between those from different cases in the latent space. Typically, a sample is processed through two random augmentation methods to form a positive pair, while all other pairs within the same batch serve as negative pairs^28,40^.

In our research, multiple slit-lamp images under different conditions and modalities were collected for each MK patient. It is hypothesized that the invariant features shared across multiple images of a single patient are highly representative and play a critical role in disease diagnosis^41^. During each iteration, we randomly sample a batch of N patients, where each patient has a varying number of images captured under different conditions. For every patient, two images are randomly selected to form a positive pair, while all images from other patients are treated as negative pairs.

Let x_i_ and x_j_ represent two images from the same patient. These images are independently augmented using two random methods and subsequently encoded by a deep network f(⋅) to produce their latent vectors h_i_ and h_j_. A projection head further transforms these latent vectors into z_i_ and z_j_, which are then used to compute the contrastive loss. The contrastive loss, defined as

(1) $${\text{L}}_{\text{con}}=-\log\frac{{\text{e}}^{\mathrm{sim}\left({\text{z}}_{\text{i}},{\text{z}}_{\text{j}}\right)/\text{τ}}}{\sum_{\text{k}=1}^{2\text{N}}1_{\text{k}\neq \text{i}}e^{\mathrm{sim}\left({\text{z}}_{\text{i}},{\text{z}}_{\text{k}}\right)/\text{τ}}}$$

where sim(·,·) is cosine similarity and τ is the temperature constant. Equation 1 encourages latent features to cluster closely with those from the same patient, while pushing them farther apart from features of different patients in the latent space. This approach enables the model to learn robust and invariant representations across modalities.

### 5.6. Phase Two: Modality-Specific Model Fine-tuning

To fully leverage the distinct information contained in slit-lamp images captured under different illumination methods, we fine-tune our networks separately using images from each lighting condition, thereby constructing three modality-specific diagnostic models. These models utilize the pretrained parameters from Cross-Modality Contrastive Learning, which effectively captures modality-consistent features. The Modality-Specific Model Fine-tuning further enhances the networks' ability to learn unique representations specific to each lighting condition.

We employ Cross-Entropy as the classification objective function, defined in Equation 2, where y_i_ represents the ground truth label, p_i_ denotes the predicted probability, and C is the total number of categories. Additionally, the contrastive learning loss (Equation 1) serves as a regularization term during fine-tuning. Consequently, our complete training loss is expressed in Equation 3, combining Cross-Entropy and contrastive learning loss terms. A hyperparameter λ, set to 0.1, is used to balance these two components.


(2) $${\text{L}}_{\text{cls}}=-\sum_{\text{i}=1}^{\text{C}}{\text{y}}_{\text{i}}\log\left({\text{p}}_{\text{i}}\right)$$

(3) $${\text{L}}_{\text{finetune}}=(1-\text{λ}){\text{L}}_{\text{cls}}+\lambda {\text{L}}_{\text{con}}$$

### 5.7. Phase Three: Multi-Modality Feature Ensemble

A simple approach to determining the final diagnosis from the multiple image-specific predictions generated by our models for each patient is to use a majority voting mechanism. However, this approach may be suboptimal as it fails to integrate information from images across different modalities comprehensively^42,43^. To address this limitation, we designed an MLP to refine the final predictions.

For each input image, we extract the feature vector from the layer preceding the classification head of the corresponding modality-specific network. For each modality, we compute the mean feature vector by averaging across all available images. The resulting vectors from three modalities are concatenated to form a 3×D feature matrix, where D denotes the dimensionality of the feature vectors. This matrix is then fed into an MLP, which integrates information from all modalities to generate the final prediction. We adopted the standard cross-entropy loss (Equation 2) to train the MLP classifier.

As noted, in clinical practice, the number of images per modality varies across patients, and some may lack images from one or more modalities entirely. In such cases, the corresponding average feature vectors are initialized as zero vectors. This design enables our framework to flexibly accommodate varying numbers of image inputs per patient while ensuring stability in prediction.

### 5.8. Model Interpretation using Grad-CAM

To gain deeper insight into the decision-making process of our models, we utilized Gradient-weighted Class Activation Map (Grad-CAM)^35^ on test set images. Grad-CAM propagates gradients back to the final convolutional layer to generate heatmaps that visualize the regions the network focuses on during classification. In these heatmaps, redder areas represent regions that the model considers more important for its decision-making process.

### 5.9. Implementation Details

We randomly split the dataset into five subject-wise groups and performed five-fold cross-validation for all experiments. All backbone networks — ResNet-50^31^, DenseNet-121^32^, SwinV2-T^33^ and PVT_v2_b1^34^ — were initialized with pretrained weights from ImageNet-1k^44^. All input images were preprocessed by applying limbus-based cropping using masks generated from our previous work^30^, followed by resizing to 256×256 pixels.

We employed the Adam^45^ optimizer with a weight decay of 1e−3. The learning rate was set to 3e−5 during Phase One, and 1e−5 during Phases Two and Three. The training epochs were 150 in Phase One, 100 in Phase Two, and 50 in Phase Three. To ensure a stable training process, we adopted a linear warm-up followed by a polynomial decay learning rate schedule. The batch size was set to 32. All models were implemented using PyTorch 2.5.0^46^ and trained on a single NVIDIA GeForce RTX 4090 GPU. Each model required approximately 6 GB of GPU memory. The complete three-phase training pipeline took approximately 7 hours. During inference, the proposed framework generated a patient-level prediction in approximately 50ms on average. Upon acceptance, the source code is available at https://github.com/yqwang01/TPMKA, and access to the dataset will be provided upon reasonable email request and subject to appropriate approvals and data-use agreements with University of Michigan.

### 5.10. Evaluation Metrics

We evaluated our model's performance using F1-score and accuracy, as formulated in Equations 4 and 5. Here, TP, TN, FP, and FN represent true positives, true negatives, false positives, and false negatives, respectively. The F1-score, defined as the harmonic mean of precision and recall, is particularly valuable for assessing model robustness in imbalanced datasets. To ensure a comprehensive evaluation, we reported both class-specific F1-scores and the overall average F1-score. Additionally, we computed the area under the receiver operating characteristic curve (AUC) to quantify the effectiveness of the model’s decision-making.


(4) $$\text{Accuracy}=\frac{\text{TP}+\text{TN}}{\text{TP}+\text{TN}+\text{FP}+\text{FN}}$$

(5) $$\text{F1-score}=2\frac{\text{Precision}\;*\;\text{Recall}}{\text{Precision}+\text{Recall}}=\frac{2\text{TP}}{2\text{TP}+\text{FP}+\text{FN}}$$

## Figures and Tables

**Table 1.** The quantitative performance evaluation of our framework and other baselines. B – Blue Light, ScS – Sclerotic Scatter, W – White Light. Mean scores over folds are presented. The standard deviation is in parentheses.

Method	Modality	Fungal	F1 Score	Average	Accuracy
			Bacterial		
ResNet-50^31^	B	88.0% (0.01)	66.8% (0.07)	77.38% (0.03)	82.58% (0.01)
	ScS	87.8% (0.01)	71.8% (0.05)	79.65% (0.02)	82.91% (0.01)
	W	87.0% (0.02)	73.0% (0.04)	79.87% (0.02)	82.46% (0.02)
	Simple Voting	87.5% (0.01)	72.9% (0.05)	80.20% (0.02)	83.04% (0.01)

DenseNet-121^32^	B	88.4% (0.01)	66.8% (0.08)	77.51% (0.04)	82.65% (0.02)
	ScS	87.4% (0.02)	71.0% (0.08)	79.12% (0.04)	82.45% (0.02)
	W	87.0% (0.01)	73.4% (0.03)	80.16% (0.01)	82.64% (0.01)
	Simple Voting	86.9% (0.01)	71.9% (0.06)	79.38% (0.03)	82.25% (0.01)

SwinV2-T^33^	B	88.4% (0.01)	69.9% (0.05)	79.14% (0.03)	83.26% (0.01)
	ScS	88.3% (0.01)	72.3% (0.08)	80.28% (0.04)	83.69% (0.02)
	W	88.0% (0.01)	76.3% (0.03)	82.13% (0.01)	84.17% (0.01)
	Simple Voting	88.7% (0.01)	76.1% (0.05)	82.39% (0.02)	84.74% (0.01)

DeepIK^23^	B	87.3% (0.01)	68.5% (0.05)	77.91% (0.02)	82.03% (0.01)
	ScS	87.6% (0.01)	72.6% (0.07)	80.06% (0.04)	83.04% (0.02)
	W	86.6% (0.01)	74.2% (0.04)	80.41% (0.02)	82.52% (0.01)
	Simple Voting	87.9% (0.01)	75.1% (0.06)	81.48% (0.03)	83.83% (0.02)

PVT_v2_b1^34^	B	88.2% (0.01)	68.8% (0.05)	78.47% (0.02)	82.93% (0.01)
	ScS	88.6% (0.01)	73.4% (0.06)	80.98% (0.03)	84.15% (0.01)
	W	86.8% (0.02)	73.7% (0.03)	80.27% (0.02)	82.52% (0.02)
	Simple Voting	88.9% (0.01)	76.7% (0.04)	82.80% (0.02)	85.05% (0.01)

Ours	B	88.4% (0.01)	69.1% (0.08)	78.76% (0.04)	83.28% (0.02)
	ScS	88.9% (0.01)	75.4% (0.06)	82.12% (0.03)	84.81% (0.01)
	W	88.0% (0.01)	76.7% (0.03)	82.36% (0.02)	84.23% (0.01)
	Ensemble	**89.0% (0.01)**	**80.0% (0.03)**	**84.46% (0.01)**	**85.84% (0.01)**

**Table 2.** The ablation study on the impact of Cross-Modality Contrastive Learning (CMCL). Mean scores over folds are presented. The standard deviation is in parentheses.

Backbone	Modality	CMCL	Fungal	F1 Score	Average	Accuracy
				Bacterial		
SwinV2-T^33^	B	√	88.4% (0.01)	69.9% (0.05)	79.14% (0.03)	83.26% (0.01)
			**89.0%** (0.00)	**71.4%** (0.05)	**80.16%** (0.03)	**83.95%** (0.01)

	ScS	√	88.3% (0.01)	72.3% (0.08)	80.28% (0.04)	83.69% (0.02)
			**89.0%** (0.01)	**74.2%** (0.06)	**81.70%** (0.03)	**84.67%** (0.01)

	W	√	88.0% (0.01)	76.3% (0.03)	82.13% (0.01)	84.17% (0.01)
			**88.4%** (0.01)	**76.8%** (0.04)	**82.56%** (0.02)	**84.54%** (0.02)

	Ensemble	√	89.0% (0.01)	79.8% (0.01)	84.35% (0.01)	85.77% (0.01)
			**89.0%** (0.01)	**79.8%** (0.01)	**84.40%** (0.01)	**85.83%** (0.01)

PVT_v2_b1^34^	B	√	88.2% (0.01)	68.8% (0.05)	78.47% (0.02)	82.93% (0.01)
			**88.4%** (0.01)	**69.1%** (0.08)	**78.76%** (0.04)	**83.28%** (0.02)

	ScS	√	88.6% (0.01)	73.4% (0.06)	80.98% (0.03)	84.15% (0.01)
			**88.9% (0.01)**	**75.4% (0.06)**	**82.12%** (0.03)	**84.81%** (0.01)

	W	√	86.8% (0.02)	73.7% (0.03)	80.27% (0.02)	82.52% (0.02)
			**88.0%** (0.01)	**76.7%** (0.03)	**82.36%** (0.02)	**84.23%** (0.01)

	Ensemble	√	89.2% (0.01)	79.2% (0.05)	84.12% (0.03)	85.71% (0.02)
			89.0% (0.01)	**80.0% (0.03)**	**84.46% (0.01)**	**85.84% (0.01)**

**Table 3.** The ablation study on the impact of metadata usage, cropping limbus, and contrastive learning regularization in fine-tuning. Mean scores over folds are presented. The standard deviation is in parentheses.

Metadata Usage	Cropping Limbus	CL Reg	Fungal	F1 Score	Average	Accuracy
				Bacterial		
	
√		√	88.2% (0.01)	77.8% (0.05)	83.04% (0.03)	84.80% (0.02)
	√		88.8% (0.00)	78.2% (0.03)	83.42% (0.02)	85.17% (0.01)
√	√		88.8% (0.00)	79.2% (0.03)	84.06% (0.01)	85.47% (0.01)
√	√	√	**89.0%** (0.01)	**80.0%** (0.03)	**84.46%** (0.01)	**85.84%** (0.01)

**Table 4.** Class distributions of the original dataset and the separately curated balanced dataset.

Site	Fungal	Original Dataset	All	Fungal	Balanced Dataset	All
		Bacterial			Bacterial	
India	961	211	1,172	87	87	174
US	87	386	473	87	87	174
Total	1,048	597	1,645	174	174	348

**Table 5.** Site-specific performance evaluation. Mean scores over folds are presented. The standard deviation is in parentheses.

Dataset	Testing Site	Method	Fungal	F1 Score	Average	Accuracy
				Bacterial		
Original	Overall	DeepIK^23^	87.9% (0.01)	75.1% (0.06)	81.48% (0.03)	83.83% (0.02)

		PVT_v2_b1^34^	88.9% (0.01)	76.7% (0.04)	82.80% (0.02)	85.05% (0.01)

		Ours (No Location)	88.7% (0.01)	78.7% (0.02)	83.68% (0.01)	85.29% (0.01)

		Ours	89.0% (0.01)	80.0% (0.03)	84.46% (0.01)	85.84% (0.01)

	India	DeepIK^23^	91.8% (0.01)	42.1% (0.11)	66.95% (0.06)	85.67% (0.02)

		PVT_v2_b1^34^	92.2% (0.01)	39.8% (0.12)	65.97% (0.07)	86.18% (0.02)

		Ours (No Location)	91.6% (0.02)	46.0% (0.11)	68.79% (0.06)	85.52% (0.03)

		Ours	91.6% (0.01)	51.9% (0.03)	71.79% (0.02)	85.79% (0.02)

	US	DeepIK^23^	31.0% (0.12)	87.3% (0.04)	59.16% (0.07)	78.83% (0.06)

		PVT_v2_b1^34^	45.9% (0.13)	89.6% (0.04)	67.77% (0.08)	82.72% (0.06)

		Ours (No Location)	37.7% (0.13)	90.9% (0.03)	64.31% (0.07)	84.25% (0.05)

		Ours	43.0% (0.10)	91.6% (0.03)	67.27% (0.06)	85.44% (0.05)

Resampling	Overall	DeepIK^23^	86.4% (0.01)	75.4% (0.06)	80.88% (0.03)	82.67% (0.01)

		PVT_v2_b1^34^	86.7% (0.02)	76.0% (0.06)	81.34% (0.04)	82.98% (0.03)

		Ours	87.2% (0.02)	78.8% (0.03)	83.03% (0.02)	84.19% (0.02)

	India	DeepIK^23^	90.3% (0.01)	54.0% (0.06)	72.18% (0.03)	84.09% (0.01)

		PVT_v2_b1^34^	90.4% (0.02)	56.2% (0.03)	73.29% (0.02)	84.24% (0.03)

		Ours	90.0% (0.03)	57.4% (0.07)	73.74% (0.05)	83.95% (0.04)

	US	DeepIK^23^	39.9% (0.11)	86.6% (0.05)	63.23% (0.07)	78.36% (0.07)

		PVT_v2_b1^34^	48.2% (0.11)	86.5% (0.07)	67.34% (0.09)	79.11% (0.10)

		Ours	49.6% (0.10)	90.5% (0.03)	70.06% (0.06)	84.12% (0.05)

Balanced	Overall	DeepIK^23^	70.9% (0.04)	77.1% (0.03)	73.96% (0.03)	74.43% (0.03)

		PVT_v2_b1^34^	70.3% (0.06)	75.3% (0.04)	72.81% (0.04)	73.29% (0.03)

		Ours	74.8% (0.04)	76.1% (0.06)	75.44% (0.05)	75.59% (0.05)

	India	DeepIK^23^	75.0% (0.10)	80.2% (0.04)	77.64% (0.06)	78.63% (0.05)

		PVT_v2_b1^34^	72.0% (0.08)	77.6% (0.06)	74.77% (0.06)	75.57% (0.06)

		Ours	79.8% (0.08)	79.6% (0.11)	79.70% (0.09)	80.24% (0.09)

	US	DeepIK^23^	64.7% (0.07)	74.1% (0.03)	69.40% (0.05)	70.24% (0.04)

		PVT_v2_b1^34^	67.8% (0.06)	72.2% (0.05)	70.00% (0.03)	70.70% (0.03)

		Ours	69.5% (0.09)	73.2% (0.07)	71.36% (0.08)	71.52% (0.08)

**Table 6.** Details of our dataset distribution of MK subtype classification. F – Fungal, B – Bacterial.

Site	Patient Count			Blue Light Image			Sclerotic Scatter Image			White Light Image		
	F	B	All	F	B	All	F	B	All	F	B	All
India	961	211	1,172	3,546	778	4,324	3,644	847	4,491	3,653	805	4,458
US	87	386	473	208	930	1,138	206	925	1,131	288	1,328	1,616
Overall	1,048	597	1,645	3,754	1,708	5,462	3,850	1,772	5,622	3,941	2,133	6,074

**Table 7.** Details of metadata information in our dataset. logMAR, logarithm of the Minimum Angle of Resolution.

Attribute Name	Description
Location	The location where the patient received the diagnosis. Possible values: India, US.
Trauma	Indicates whether the patient has experienced trauma to the eye. Possible values: Yes, No, Unknown.
Organic Matter	Indicates whether the patient’s eye has been exposed to organic matter. Possible values: Yes, No, Unknown.
Water Exposure	Indicates whether the patient’s eye has been exposed to water. Possible values: Yes, No, Unknown.
Contact Lens Usage	Indicates whether the patient wears contact lenses. Possible values: Yes, No, Unknown.
Best Corrected Visual Acuity	The patient’s visual acuity in the affected eye, in logMAR units, with the use of any corrective lenses or with manifest refraction.
Uncorrected Visual Acuity	The patient’s visual acuity in the affected eye, in logMAR units, without corrective lenses.

## Data Availability

The datasets generated and/or analysed during the current study are not publicly available due to institutional patient privacy restrictions and data-use agreements, but may be made available from the corresponding author upon reasonable email request and subject to appropriate approvals and data-use agreements with University of Michigan.

## Code availability

The custom code used for model training and evaluation is publicly available at https://github.com/yqwang01/TPMKA and in the supplementary materials.
